# The discovery of berberine erythrocyte-hemoglobin self-assembly delivery system: a neglected carrier underlying its pharmacokinetics

**DOI:** 10.1080/10717544.2022.2036870

**Published:** 2022-03-11

**Authors:** Qiuxia Yu, Minhua Li, Hanbin Chen, Lieqiang Xu, Juanjuan Cheng, Guoshu Lin, Yuhong Liu, Ziren Su, Xiaobo Yang, Yucui Li, Jiannan Chen, Jianhui Xie

**Affiliations:** a The Second Clinical College of Guangzhou, University of Chinese Medicine, Guangzhou, China; b School of Pharmaceutical Sciences, Guangzhou University of Chinese Medicine, Guangzhou, China; c The First Affiliated Hospital of Chinese Medicine, Guangzhou University of Chinese Medicine, Guangzhou, China; d The Second Affiliated Hospital of Guangzhou University of Chinese Medicine, Guangzhou, China; e State Key Laboratory of Dampness Syndrome of Chinese Medicine, The Second Affiliated Hospital of Guangzhou University of Chinese Medicine, Guangzhou, China; f Guangdong Provincial Key Laboratory of Clinical Research on Traditional Chinese Medicine Syndrome, Guangzhou, China

**Keywords:** Berberine, erythrocyte, hemoglobin, self-assembly system, pharmacokinetics

## Abstract

Berberine (BBR) has extremely low concentration and high tissue distribution. However, current pharmacokinetic studies predominantly focus on its concentration in plasma, which could hardly make a comprehensive understanding of its pharmacokinetic process. This study made a pioneering endeavor to explore the erythrocyte-hemoglobin (Hb) self-assembly system of BBR by exploring the interaction of BBR with erythrocyte and the combination of BBR with Hb. Results showed that BBR had a low bioavailability (*C_0_* = 2.833 μg/mL via intravenous administration of 2.5 mg/kg BBR and *C_max_* = 0.260 μg/mL via oral administration of 400 mg/kg BBR). Besides, BBR achieved higher concentrations in erythrocytes than plasma, and the erythrocytes count and Hb content were significantly decreased after intravenous administration. Hemolysis rate indicated the BBR-erythrocyte system (with 2% erythrocytes) was relatively stable without hemolysis at the concentration of 1.00 mg/mL. And the maximum percentage of drug loading was 100% when the BBR-erythrocyte concentration was 0.185 μg/mL. Furthermore, incubation of BBR and erythrocytes resulted in internalization of the erythrocyte membrane and the formation of intracellular vacuoles. The thermodynamic parameters indicated that the binding process of bovine hemoglobin (BHB) and BBR was spontaneous. UV-vis absorption spectra, synchronous fluorescence, circular dichroism and Raman spectra collectively indicated that BBR showed strong binding affinity toward BHB and affected the molecular environment of residues like tryptophan and tyrosine in BHB, resulting in the conformational changes of its secondary and tertiary structure. Molecular docking indicated BBR interacted with Arg-141 residue of BHB via hydrogen bond with the bond length of 2.55 Å. The ΔG value of the BHB-BBR system was −31.79 kJ/mol. Molecular dynamics simulation indicated the root mean square derivation of BBR-BHB was <0.025 nm, suggestive of stable conformation. Cumulatively, there was an erythrocyte-Hb self-assembled drug delivery system after oral or intravenous administration of BBR, which conceivably gained novel insight into the discrepancy between the extremely low plasma concentration and relatively high tissue concentration of BBR.

## 1. Introduction

Berberine (BBR), is the main active ingredient of medicinal plants like *Phellodendron chinense* Schneid. and *Coptis chinensis* Franch., possesses multiple pharmacological effects, and has gained widespread clinical applications, including anti-diabetic (Cicero and Baggioni, 2016), anti-inflammatory (Zou et al., 2017; Fu et al., 2021), anti-Alzheimer's disease (Lau et al., 2001; Cai et al., 2016; Hou et al., 2020), anti-cardiovascular (Feng et al., 2019), anti-cancer (Ortiz et al., 2014; Sun et al., 2019; Gu et al., 2020), and anti-hyperuricaemia (Xu et al., 2021) activities and so on.

The efficacy of drugs is largely influenced by its pharmacokinetic action. Due to the poor intestinal absorption and rapid fist-pass metabolism, BBR has extremely low oral bioavailability (less than 1%), which limits its wide applications (Liu et al., 2010; Chen et al., 2011). However, recent studies have suggested that BBR and its metabolisms were widely distributed in various tissues, especially the liver, in which the average concentration of BBR was approximately 10–30 times higher than that in plasma (Yan et al., 2009; Tan et al., 2013; Yu et al., 2017). Hence, it seems that there is a contradiction between the extremely low plasma concentration and relatively high tissue concentration of BBR.

Most current pharmacokinetics studies only focus on the concentration of free drugs in plasma (Zuo et al., 2006; Gong et al., 2014), while a study on the role of other major components in blood like erythrocyte is rare and insufficient. Indeed, among the cellular constituents of blood, the erythrocytes represent, by far, the largest population both in number and cell size. Erythrocytes have a remarkably long life span and a widespread circulation throughout the body, and have become a natural drug carrier with incomparable superiorities of biocompatibility and biodegradability (Szumiło, 2013; Koleva et al., 2020). Erythrocytes can quickly identify the targeted delivery including drug molecule, transport that to the target site and accumulate in the liver (Fan et al., 2012b). Hence, erythrocytes have currently been considered as an excellent site-targeted delivery system (Hamidi et al., 2007).

Hemoglobin (Hb), the most abundant protein in erythrocytes, can reversibly bind with many endogenous and exogenous molecules (Wang et al., 2007), and acts as a drug carrier for effective delivery to the required physiological site for drug therapy (Hazra et al., 2013). BBR was believed to be a drug with a high protein binding rate for its pharmacokinetic characteristics. Our recent work has suggested BBR was mainly presented and transported in the proteins-bound form and exerted much stronger binding interaction with Hb than plasma, and BBR binding to Hb was a basis for the accumulation of BBR in the blood (Chen et al., 2021).

Since the concentration of BBR in the liver was about 10-fold higher than that in the plasma, the pharmacokinetics of BBR accorded with the characteristic of distribution and delivery of erythrocytes extremely. BBR can be easily adsorbed onto the erythrocytes, which promotes the uptake of BBR by erythrocytes. As a corollary, from the perspective of the drug system, it was hypothesized that erythrocyte might represent a natural reservoir for BBR, and BBR and erythrocytes could act as a self-assembled drug delivery system and be delivered to the target organs through a special system and mechanism after entering the bloodstream to improve its pharmacological response.

As a follow-up study and to test our hypothesis, in the present work, the pioneering endeavor has been devoted to investigating the mechanism of the possible erythrocyte-hemoglobin (Hb) self-assembled drug delivery system on BBR including the interaction of BBR and erythrocytes as well as the binding mechanism of BBR and Hb. The findings revealed erythrocyte might represent an important compartment and a natural reservoir for BBR transport in the blood stream, and the mechanism of transport of BBR based on the view of interaction with Hb and provided enlightenment for the study on the transport of other natural components using erythrocyte-Hb self-assembly drug delivery system.

## 2. Materials and methods

### 2.1. Animal

Sprague Dawley (SD) rats (male, 200 ± 20 g) were purchased from the Laboratory Animal Center of Guangzhou University of Chinese Medicine (GZUCM, Guangzhou, China). Before the experiment, all the rats were kept in the animal house under artificial lighting of 12 h light/dark cycle, with 25–28 °C and 60%–65% humidity. They were fed with laboratory regular diet, and sterilized water was freely consumed. The experimental procedures were carried out in accordance with the guideline of the Institutional Ethics Committee of Guangzhou University of Chinese Medicine. The ethical approval for the animal experiments was performed in accordance with the NIH Guidelines for the Care and Use of Laboratory Animals (EthicNo.2016047).

### 2.2. Chemicals and materials

Deionized water was obtained from a Millipore Milli-Q Academic water purification system (Millipore, Bedford, MA, U.S.A.). Bovine hemoglobin was obtained from Sigma Aldrich (St. Louis, MO, U.S.A.). BBR was purchased from Chengdu pureChem-Standard Co., Ltd. (Chengdu, China, with a purity ≥ 98%). The internal standard (I.S.), piperine, was purchased from the National Institute for the Control of Pharmaceutical and Biological Products (Beijing, China, the purity ≥98%). HPLC-grade acetonitrile was purchased from Guangzhou Lubex Biological Technology Co., Ltd. (Guangzhou, China). Electron microscope fixing solution (G1102) was obtained from Servicebio (Wuhan, China). The purity of heparin sodium was ≥ 99%. NaCl, HCl, Tris and other reagents were all of the analytical purity. Tris-HCl buffer solution (0.05 mol/L Tris-HCl, 0.15 mol/L NaCl, pH 7.4) was used to dissolve all samples.

The Shim LC-20CE liquid chromatography system was used for *in vitro* analysis and the chromatographic separation of BBR has proceeded on Phenomenex Luna C18 column (150 mm × 4.6 mm, 5 μm) at room temperature. The interaction between bovine hemoglobin (BHB) and BBR was detected by MADLITOF/TOF Analyzer (California, USA). The content of Hb was calculated by hemoglobin colorimetric assay (Leagene, China).

Ultra-thin microtome (Leica, Germany) and Diamond slicer (Diamond, Germany) was carried out to make slices. The interaction between BHB and BBR was detected by MADLITOF/TOF Analyzer (California, USA). The content of Hb was calculated by hemoglobin colorimetric assay (Leagene, China). All fluorescence spectra were measured by the F-4600 Fluorescence Spectrophotometer (Hitachi, Japan). The Raman spectrum was measured by a Raman spectrophotometer (VERTEX70, Bruker, Germany) at a resolution ≤ 0.5 cm^−1^. The widths of both the excitation slit and the emission slit were set at 5 nm. The scan speed was 1200 nm/min. The pH was measured by a pHs-3C acidity meter (Pengshun, Shanghai, China). The molar ratio (1:1) of BHB: BBR was used for the measurement. BHB and BBR were dissolved in Tris-HCl (pH 7.4), and the solutions were freeze-dried into powder.

### 2.3. Bioavailability study of BBR via intravenous and oral administration

Sixteen SD rats were randomly divided into two groups (*n* = 8) and received a single intragastrical administration (i.g.) of BBR at the dose of 400 mg/kg and intravenous injection (i.v.) at 2.5 mg/kg dose, respectively. Blood samples (0.5 mL) were obtained from the retroorbital sinus at 0.083, 0.167, 0.25, 0.5, 1, 2, 4, 8 and 24 h after administration for i.v. group and at 0.083, 0.167, 0.25, 0.5, 1, 2, 4, 8 and 24 h for i.g. groups. The plasma samples were processed with a protein precipitation method using acetonitrile and the concentration of free drug was analyzed by HPLC (Li et al., 2016).

### 2.4. Peripheral blood cells in rats

After 48 SD rats were fed adaptively for a week, they were randomly divided into the control group and i.v. group (24 rats per group). Then they were further divided into 5, 30, and 120 min subgroups (*n* = 8). The SD rats in the i.v. group were intravenously administrated with 2.5 mg/kg BBR, while the control group was intravenously administrated with 1 mL 0.9% physiological saline solution. The blood samples were collected from the retroorbital sinus to K_2_EDTA-coated tubes, and used for the evaluation of haematological indicators including erythrocyte count, white blood cell (WBC) count, platelet count (PLT) and Hb.

### 2.5. Erythrocytes/plasma partition coefficient of BBR *in vivo*

After six SD rats were intravenously administrated with BBR, blood samples were obtained from posterior orbital venous plexus to a heparinized tube at 5, 30, and 120 min, respectively, and then centrifuged at 2500 rpm for 10 min. After centrifugation, the upper layer (plasma) was collected, while the lower layer (blood cells) was washed three times with physiological saline and centrifuged for 5 min at 3000 rpm, and then resuspended in an appropriate volume of physiological saline, which was obtained as erythrocytes to perform the following procedure:

Piperine was added into the obtained plasma as the internal standard substance and then the samples were mixed with acetonitrile to precipitate protein. The mixtures were centrifuged at 8000 rpm for 10 min. The supernatants were collected and dried. The eluates were concentrated and re-dissolved for analysis.

The obtained erythrocytes were mixed with piperine and HC, and placed in a water bath at 60 °C for 6 h and then centrifuged at 12,000 rpm for 10 min. The supernatant was obtained and injected through a solid-phase extraction to separate drugs and impurities. The filtrate was collected, dried, and then re-dissolved with 200 μL acetonitrile for HPLC analysis (Chen et al., 2021).

### 2.6. Preparation of erythrocyte *in vitro*

The whole blood was collected from rats by orbital plexus vein and transferred into a blood-collection tube containing ethylene diamine tetraacetic acid (EDTA) as an anticoagulant. The whole blood was centrifuged at 3000 rpm for 10 min. The plasma and the buffy coat in the supernatant were removed by aspiration, and erythrocytes were washed three times in cold phosphate buffer saline (PBS) and centrifuged for 10 min at 3000 rpm, and then resuspended in an appropriate volume of PBS.

### 2.7. Hemolysis rate

The hematocrite of washed erythrocytes was adjusted by PBS to 2%. In 5-mL Eppendorf tubes, 1 mL of 2% erythrocytes was incubated with 1 mL of different concentrations of BBR hydrochloride at 37 °C for 2 h. The final concentrations of BBR hydrochloride in reaction system were 0.125, 0.250, 0.500, 1.000, 1.333, 1.500, 1.600 mg/mL, respectively. One mL of 2% erythrocytes incubated with 1 mL saline solution served as the positive group while 1 mL 2% erythrocytes incubated with 1 mL distilled water served as the negative one. After incubation, the erythrocytes suspension was centrifuged at 3000 rpm for 10 min. Then 150 μL supernatant was added to the 96-well plate. Three parallel wells were set for each group, and the absorbance at 545 nm of each sample well was measured by a microplate reader. The imaging system was employed to observe whether BBR induced hemolysis. The hemolysis rate was calculated according to the following formula:

$$\text{Hemolysis rate }(\%)\;=\;[({\mathrm{OD}}_{\mathrm{sample}}-{\mathrm{OD}}_{\mathrm{negative}})/({\mathrm{OD}}_{\mathrm{positive}}\;-{\mathrm{OD}}_{\mathrm{negative}})]\;\times 100\%$$

### 2.8. Berberine loading procedures

In order to determine the effect of BBR concentration on loading efficiency, different concentrations (1.000, 0.100, 0.010 and 0.001 M) of BBR were comparatively used at 37 °C for fixed incubation time (2 h) to obtain the more suitable concentration for the loading process. The hematocrite of washed erythrocytes was adjusted by PBS to 30%. In the 5-mL Eppendorf tubes, 1 mL of 30% erythrocytes was added to 1 mL PBS containing the known concentration of the agent. The final concentrations of BBR in the reaction system were 0.500, 0.050, 0.005, 0.0005 M (185, 18.5, 1.85 and 0.185 μg/mL), respectively. The mixtures were gently mixed to avoid hemolysis and incubated for 2 h at 37 °C. After incubation, the erythrocytes suspension was centrifuged for 10 min at 3000 rpm and the supernatant was collected.

In order to verify the binding system, the supernatant was analyzed by quantifying the amount of unbound BBR through HPLC. In order to offset this effect, an internal standard method was employed in this study and piperine was chosen as the internal standard substance (200 μg/mL). Different known concentrations (500.00, 100.00, 20.00, 4.00, 0.80, 0.16 μg/mL) of BBR and piperine were used to create the standard curve. The peak area of each concentration with the concentration of piperine was measured 3 times. And then, the calibration curves were prepared using linear regression of known drug concentration versus peak area. The unknown concentration of BBR was analyzed by the regressed equation: *y* = a(x) + b. While y is the peak area ratio of BBR and piperine, a is the slope, b is the y-intercept, and x is the concentration of BBR.

After incubation, acetonitrile was added to the different incubated solutions for protein precipitation. The mixtures were centrifuged at 8000 rpm for 10 min. The supernatants were collected and dried. The eluates were concentrated and re-dissolved for analysis. The loading efficiency was calculated according to the following formula:

$$\text{Loading efficiency }(\%)\;=\;[(M_{\text{original drug}}-M_{\text{drug after incubation}})/M_{\text{original drug}}]\;\times 100\%$$

### 2.9. Transmission electron microscopy

A transmission electron microscope was used to evaluate the morphological differences between normal erythrocytes and BBR-loaded erythrocytes. For the electron microscopic studies, 30% erythrocytes were treated with PBS for 2 h at 37 °C as the control group, while 30% erythrocytes in the experimental group were treated with 0.5 M BBR at 37 °C for 2 h. After centrifugation, the supernatant was removed and the cells were fixed in an electron microscope fixing solution at 4 °C for 2–4 h. Then cells were wrapped in 1% agarose and rinsed 3 times for 15 min in 0.1 M phosphoric acid buffer PBS (pH 7.4).

The samples were post-fixed in 1% osmium tetroxide at indoor temperature (20 °C) for 2 h and rinsed with 0.1 M PBS (pH 7.4) 3 times for 15 min each in 0.1 M phosphoric acid buffer PBS (pH 7.4). After this, the samples were dehydrated using a graded ethanol series: 50, 70, 80, 90, 95, 100 and another 100% acetone for 15 min each. After permeation and embedding, appropriate blocks were selected for thin sectioning on an ultramicrotome at 60–80 nm thickness. Then the sections were double-stained with uranyl acetate and lead citrate and viewed under the transmission electron microscope.

### 2.10. Spectrum procedure

Firstly, 1.0 mL of BHB (2.0 × 10^−6 ^mol/L) was mixed with 1.0 mL of 0.05 mol/L Tris-HCl buffer (pH = 7.4). And then BHB was added with different amounts of 0.25 × 10^−3 ^mol/L stock solution of BBR with an increment of 0.25 × 10^−5 ^mol/L until 1.75 × 10^−5 ^mol/L. The absorbance of all the samples was detected by UV-vis absorption spectra (excitation at 280 nm and emission wavelengths ranging from 295 to 400 nm). The synchronous fluorescence spectra were measured through simultaneous scanning of the excitation (*λ*ex = 240 nm) and emission monochromators while maintaining a constant wavelength interval between them (Δ*λ*, 15 nm and 60 nm).

In addition, CD spectra were collected from 200 to 260 nm with three scans averaged for each CD spectrum. The molar ratio (1:1) of BHB: BBR was used for the measurement. Briefly, BHB and BBR were dissolved in Tris-HCl (pH 7.4), and the solutions were freeze-dried into powder. Raman spectra were measured from 2600 to 400 cm^−1^ using the Nd-YAG laser (1064 nm). Due to the existence of inner filter effects, the fluorescence parameter was corrected using the equation:

$$F=F_{0}\times 10^{\left(\frac{A_{\mathrm{EX}}+A_{\mathrm{EM}}}{2}\right)}$$

where *F* and *F*_0_ are the fluorescence intensity corrected and observed, respectively; *A*_EX_ and *A*_EM_ are the sums of the absorbance of samples at excitation and emission wavelengths (Chen et al., 2021).

### 2.11. Molecular docking and molecular dynamics simulation

AutoDock 4.2 program was used in the molecular docking study and the Lamarckian Genetic Algorithm (LGA) was utilized in this calculation. The crystallographic coordinate of BBR was retrieved from the Pub-Chem Database. The native structure of BHB (PDB ID: 1G09) was downloaded from the Protein Data Bank. In order to meet the requirement in the Lamarckian Genetic Algorithm, all water molecules were discarded, and hydrogen atoms were added after the calculation of Gasteiger charges. The grid size along the x-, y-, z-axes were set to 70 × 70 × 70 with a grid spacing of 0.375 Å, respectively. The grid box was located at the coordinates *x* = −1.07, *y* = 65.021, *z* = 12.094 of the protein molecule (Li et al., 2016). The AutoDocking parameters were as follows: GA population size was set as 150, a maximum number of energy evaluations equal to 250,000 and GA crossover mode of two points. The lowest binding energy conformer was searched out of 250 different conformers for the docking simulation and the resultant one was used for further analysis. The final conformations were viewed using PyMOL (http://www.pymol.org) software. Molecular dynamics (MD) simulation of BBR-BHB system was carried out with Gromacs 2018 packages using GROMOS96 43a1 force field. The PRODRG server was used to generate the ligand topology files (Zhang et al., 2000). Finally, the full system of BBR-BHB was subjected to an MD simulation run for 200 ns.

### 2.12. Statistical analysis

All the statistical calculations were performed by SPSS 19.0 software (IBM, USA) and Origin Pro 2021 (Origin Lab, USA). Data were expressed as mean ± S.E.M. Comparisons between multiple groups were made by one-way analysis of variance (ANOVA) followed by Least Significant Difference (LSD). *p* < .05 was considered as statistical significance.

## 3. Results

### 3.1. Bioavailability study of BBR via intravenous and oral administration

**Figure 1.**
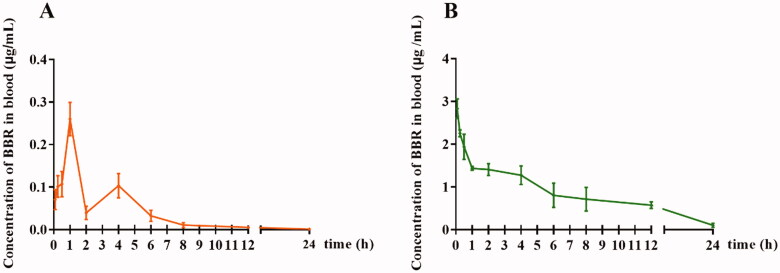
The blood concentration-time profile of free BBR. (A) Oral administration of BBR (400 mg/kg), (B) Intravenous administration of BBR (2.5 mg/kg).

As shown in Table 1 and Figure 1, the *C_0_* of BBR in rat plasma was 2.833 μg/mL after intravenous administration of 2.5 mg/kg BBR, followed by a rapid decline to baseline within 24 h. After oral administration of 400 mg/kg BBR, the maximum plasma concentration (*C_max_*) of BBR was only 0.260 μg/mL and then decreased rapidly. The *AUC_0–t_* value was 6.422 and 0.681 μg/mL × h for intravenous and oral administration, respectively. The mean elimination half-life (*t_1/2_*) of BBR following intravenous or oral administration was 3.136 and 3.885 h, respectively.

**Table 1.** Pharmacokinetic parameters of BBR after oral and intravenous administration in rats ($\bar{x}$ ± $s_{\bar{x}}$) (*n* = 8).

Parameters	Oral administration (400 mg/kg)	Intravenous administration (2.5 mg/kg)
*AUC_0–t_* (μg/mL*h)	0.681 ± 0.093	6.422 ± 0.685
*AUC_0–∞_* (μg/mL*h)	0.685 ± 0.093	7.438 ± 0.548
*MRT* (h)	4.525 ± 0.515	5.600 ± 0.561
*t*_1/2z_ (h)	3.136 ± 0.357	3.885 ± 0.389
*T*_max_ (h)	1.000 ± 0.000	–
*Clz/F* (L/h/kg)	253.89 ± 10.510	131.430 ± 2.513
*C*_max_*/C*_0_ (μg/mL)	0.260 ± 0.032	2.833 ± 0.132

Although the *AUC_0–t_* value of intravenous administration was higher than that of oral administration, the content of the free drug was still extremely low compared with that of the oral administration route. The finding indicated that in addition to gastrointestinal absorption and hepato-intestinal fist-pass metabolism, the blood environment could affect the concentration of BBR in the blood.

### 3.2. The haematological indicators in rats

**Figure 2.**
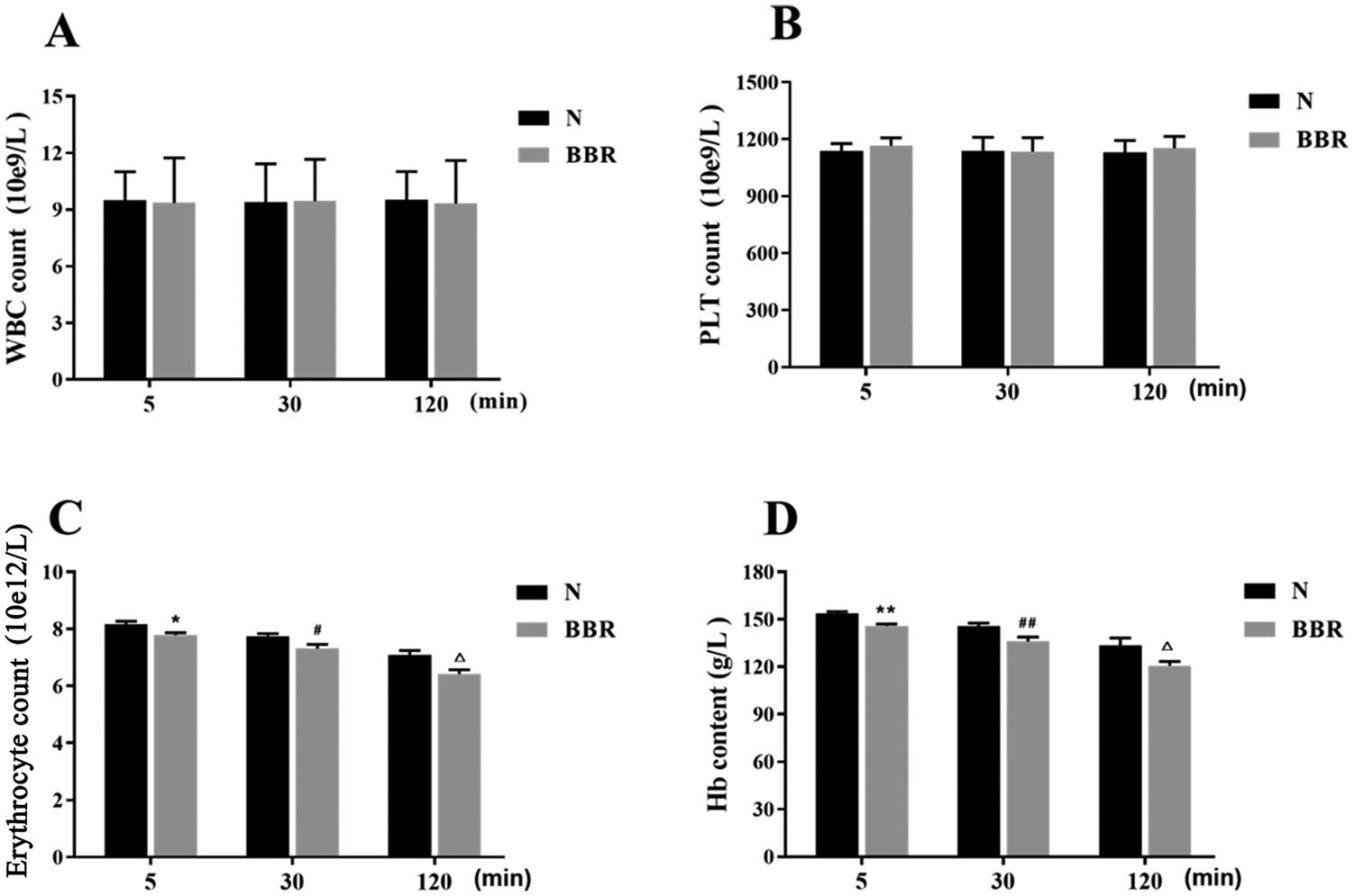
Effects of BBR on contents of WBC, erythrocyte, PLT and Hb (*n* = 8). WBC: white blood cell, PLT: platelet, Hb: hemoglobin. *p < .05, **p < .01 vs Normal 5 min; *^#^p* < .05, *^##^p* < .01 vs Normal 30 min; ^△^*p* < .05, ^△△^*p* < .01 vs Normal 120 min.

As shown in Figure 2, compared with the control group, intravenous administration of BBR for 5, 30 and 120 min had no significant effects on white blood count and platelet count (*p* > .05), whereas the erythrocytes count and Hb content were significantly reduced (*p* < .01 or *p* < .05). This finding suggested that intravenous administration of BBR would affect the levels of erythrocytes and Hb in the blood.

### 3.3. Erythrocytes/plasma partition coefficient of BBR *in vivo*

Based on the previous study, BBR could bind to the plasma and the free drug molecules in plasma partition into erythrocyte (Romański et al., 2018; Chen et al., 2021). Therefore, the erythrocytes/plasma partition coefficient (*C_e/p_*) value was calculated as the ratio of the total concentration of BBR in the erythrocyte lysate (*C_e_*) and plasma (*C_p_*). As tabulated in Table 2, the *C_e/p_* value of BBR was 1.67 ± 1.50, 0.62 ± 0.60 and ∞ at 5, 30 and 120 min, respectively. The *C_e/p_* values became smaller with the time of intravenous administration. The *C_e/p_* value was close to ∞ after intravenous administration with BBR for 2 h, potentially indicating that most of BBR entered into erythrocyte and bound with Hb.

**Table 2.** *C*_e/p_ of BBR determined in rats following intraperitoneal injection at the dose of 2.5 mg/kg.

Time (min)	Rats no.	*C_e_* (μM)	*C_p_* (μM)	*C_e/p_*
5	1	1.313	0.000	∞
	2	0.039	0.489	0.081
	3	0.000	0.000	–
	4	0.375	0.233	1.610
	5	0.862	0.233	3.700
	6	0.584	0.416	1.405
30	1	0.539	0.616	0.875
	2	0.044	0.000	∞
	3	0.000	0.000	∞
	4	0.212	0.000	∞
	5	0.026	0.282	0.090
	6	0.176	0.197	0.897
120	1	0.225	0.000	∞
	2	–	1.243	–
	3	0.000	0.000	∞
	4	0.000	0.000	∞
	5	0.000	0.000	∞
	6	0.132	0.000	∞

*C*_e_: Drug concentration in erythrocytes, *C*_p_: Drug concentration in plasma, *C*_e/p_: Erythrocytes/plasma partition coefficient. ∞: Infinity.

### 3.4. Hemolysis rate

**Figure 3.**
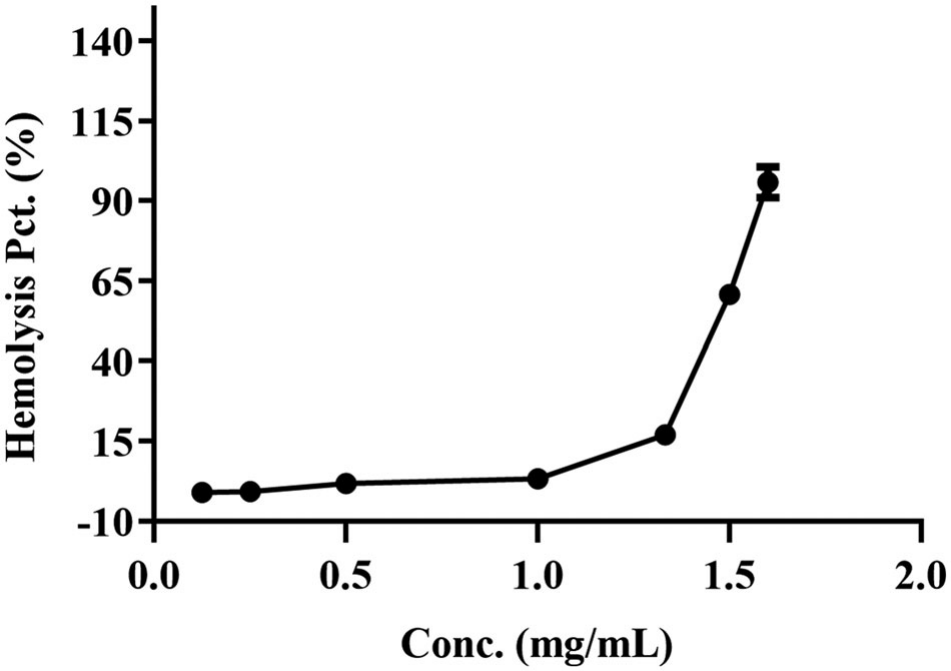
The hemolysis rate of BBR at different concentrations for 2% erythrocytes.

As shown in Figure 3, there was no hemolysis of the erythrocyte could be detected at BBR concentration below 1.00 mg/mL. When the concentration reached 1.50 mg/mL, a visible hemolysis phenomenon was observed and the hemolysis rate was 58.16%. When the concentration was 1.60 mg/mL, the hemolysis rate was up to 89.73%, which indicated that most of the erythrocytes were disrupted and the Hb was released into the plasma. The result suggested that the BBR-erythrocyte system was relatively stable without hemolysis at the concentration of 1.00 mg/mL.

### 3.5. BBR concentration on loading efficiency

**Figure 4.**
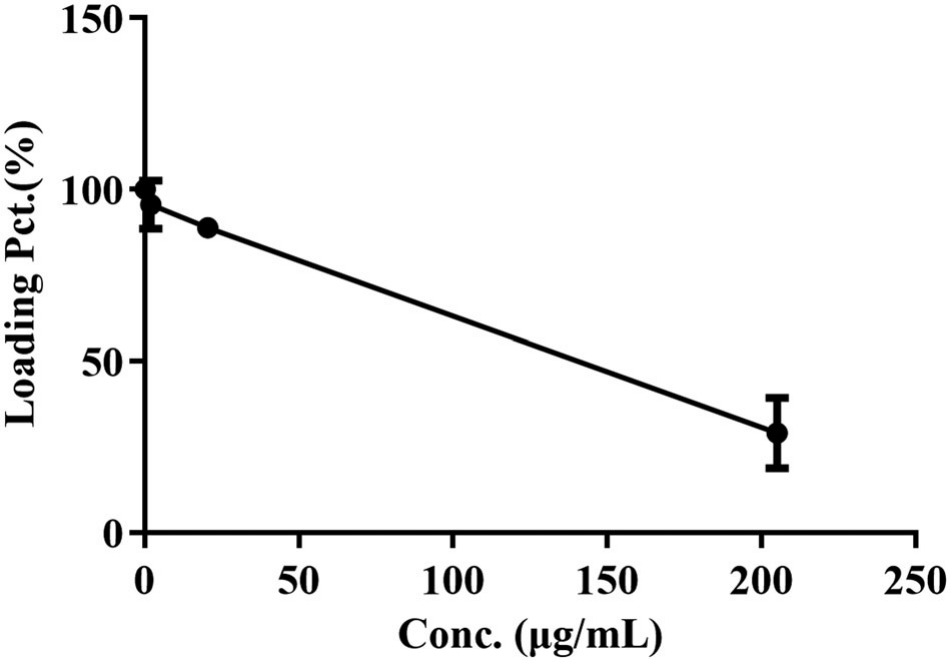
Effect of BBR concentration on 30% erythrocytes at 37 °C for 2 h.

As shown in Table 3 and Figure 4, the percentage of drug loading was significantly decreased as BBR doses increased after incubation with BBR. And the maximum percentage of drug loading was 100% when the BBR-erythrocyte concentration was 0.185 μg/mL. Hence, 0.5 M (92.500 μg/mL) BBR was selected for the following experiments.

**Table 3.** Effect of BBR concentration on 30% erythrocytes at 37 °C for 2 h ($\bar{x}$ ± $s_{\bar{x}}$) (*n* = 3).

The BBR concentration in BBR-erythrocyte system (μg/mL)	Extracellular BBR concentration after incubation (μg/mL)	Loading percentage (%)
185.000	181.843 ± 10.611	29.070 ± 4.141
18.500	2.288 ± 0.105	88.772 ± 0.410
1.850	0.115 ± 0.072	95.528 ± 2.622
0.185	0 ± 0.051	100 ± 19.84

### 3.6. Electron microscopy of berberine-induced vacuoles

**Figure 5.**
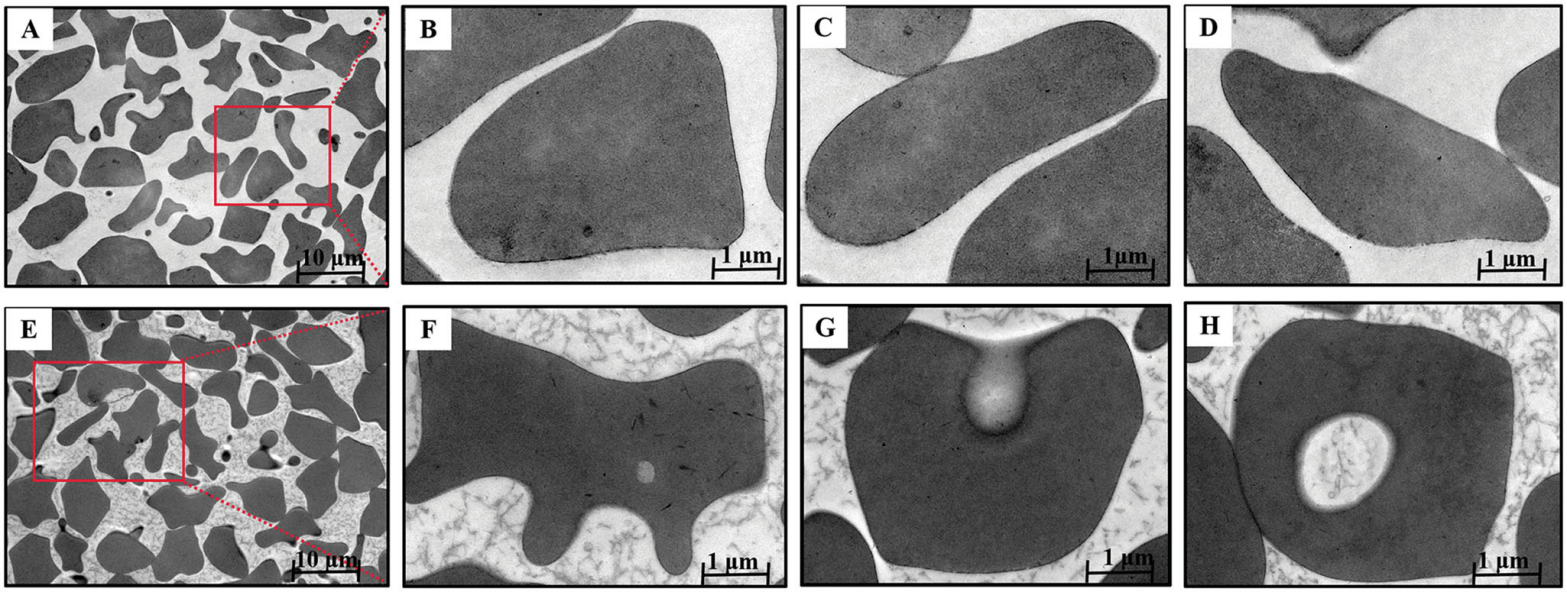
Transmission electron microscopy of BBR-loaded erythrocytes by endocytosis. Control erythrocytes (A–D) were typical biconcavediscs with high electron density, and BBR-loaded erythrocytes (E–H) were close to the circular with several discrete vacuoles. Magnification was ×1,100 and ×10,400, respectively.

The morphologic appearance of the BBR-induced vacuoles as seen by electron microscopy was shown in Figure 5. Control cells (Figure 5(A–D)) were typical biconcave discs surrounded by unit membrane without any vacuoles. Internally the cells were homogeneous and of high electron density. In contrast, as for the erythrocytes (Figure 5(E–H)) incubated with BBR *in vitro*, the cells were spheroidal with litter hemolysis and several discrete vacuoles could be actually seen inside the cells compared with normal erythrocytes. These findings suggested that BBR could interact with erythrocytes and the vacuoles of erythrocytes could be induced by BBR, facilitating the swallow-up of BBR by erythrocytes when BBR was in contact with erythrocytes in the blood.

### 3.7. Fluorescence effect of BHB by BBR

Fluorescence quenching could be used to explore the interaction between drugs and proteins, and further elucidate the interaction between small drug molecules and biological macromolecules at the molecular level. Due to the quenching mechanisms were usually classified as static and dynamic quenching, the Stern-Volmer equation was utilized to calculate the fluorescence quenching data (Wang et al., 2007):

$$\frac{F_{0}}{F}=1+K_{\mathrm{sv}}[Q]=1+k_{q}\tau_{0}[Q]$$

where *F*_0_ and *F* are the fluorescence intensities at the absence or presence of BBR after correction by Equation, respectively. *K*_sv_ is the Stern-Volmer quenching constant, while [*Q*] is the concentration of the quencher. In addition, *k*_q_ is the quenching rate constant of the binding system, and *τ*_0_ is the lifetime of fluorescence at the absence of quencher (*τ*_0_ = 10^−18 ^s) (Sousa et al., 2008).

**Figure 6.**
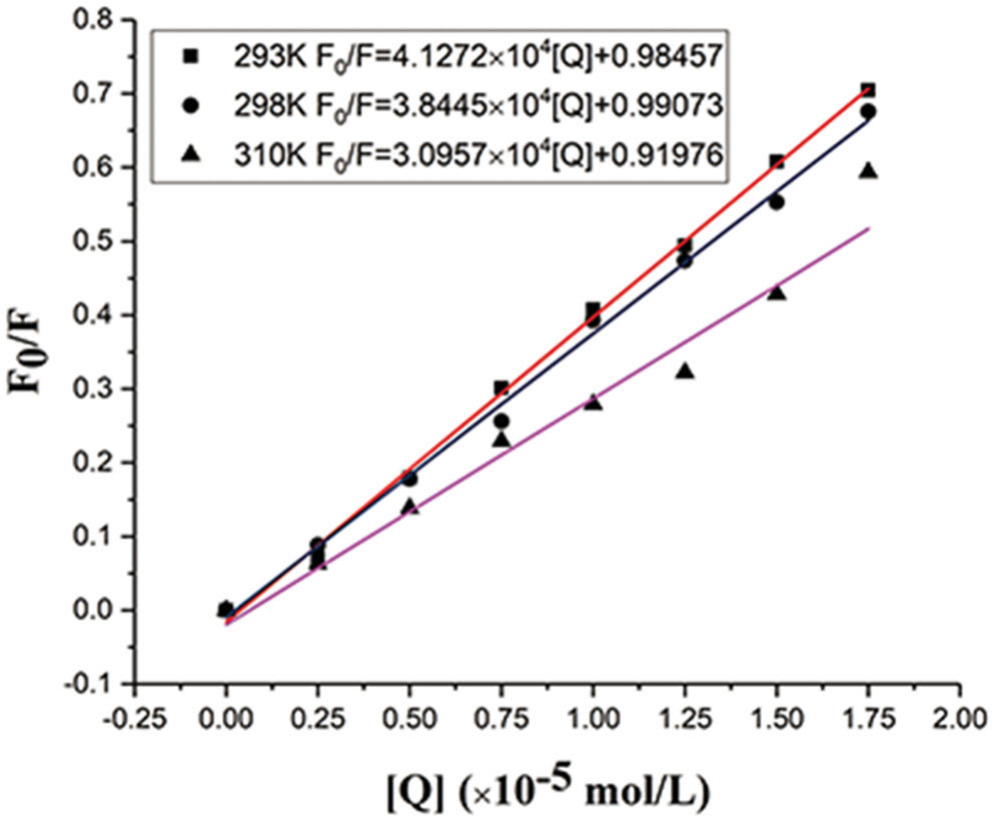
Stern-Volmer plots for the quenching of BHB by BBR (A) at different temperatures (corrected). Conditions: BHB: 2 × 10^−6 ^mol/L; pH = 7.4.

The corrected Stern-Volmer plots for the quenching of BHB by BBR and the calculated *K*_SV_ and *K*_q_ values at three different temperatures were listed in Table 4 and Figure 6. As higher temperatures may lead to smaller diffusion coefficients, the static quenching constants will decrease with increasing temperature (Zhang et al., 2000). Results showed that both *K*_SV_ and *K_q_* values deceased with the increment of temperature. The result indicated that BBR could bind with BHB and formed a BBR-BHB complex via a static quenching process.

**Table 4.** Stern-Volmer quenching constants for the interaction of BBR with BHB at three different temperatures.

Sample	*T* (K)	*K_sv_* (×10^4^ L mol^−1^)	*k_q_* (×10^12^ L mol^−1^ s^−1^)	*R* ^*a*^
BBR	293	4.1272	4.1272	0.99808
	298	3.8445	3.8445	0.99670
	310	3.0957	3.0957	0.97000

^a^R is the correlation coefficient.

### 3.8. Thermodynamic parameters and binding forces

In our earlier research, we have explored the association constants and the number of binding sites, and we have learned that in a static quenching system when a small molecule combines with a set of sites on a macromolecule, some constant can be calculated from the following equation:

$$\mathrm{lg}\left(\frac{F_{0}-F}{F}\right)=\mathrm{lgKa}+\mathrm{nlg}[Q]$$

where *F*_0_ and *F* are the fluorescence intensities in the absence and presence of BBR after correction by Equation in *Spectrum procedure*, Ka and *n* mean the association constants and the number of the sites per BHB molecule. The interrelated results were shown in our previous work (Chen et al., 2021).

A thermodynamic process is responsible for this combination since the binding constant depends on the temperature. Therefore, the values of thermodynamics were analyzed to further study the acting forces of BBR-BHB complexes. There are mainly four interaction forces between small molecule and macromolecule, including hydrophobic interaction forces, electrostatic forces, van der Waals forces and hydrogen bonds. The binding mode can be determined by the thermodynamic parameters which consist of three changes, namely enthalpy change (Δ*H*°), entropy change (Δ*S*°) and free energy change (Δ*G*°). The Δ*H*° remains as a constant when the temperature changes little.

The thermodynamic parameters were analyzed using the following equations:

$$\ln\left(\frac{{\left(K_{a}\right)}_{2}}{{\left(K_{a}\right)}_{1}}\right)=(\frac{1}{T_{1}}-\frac{1}{T_{2}})(\frac{\Delta H{}^{\circ}}{R})$$

$$\Delta G{}^{\circ}=\Delta H{}^{\circ}-T\Delta S{}^{\circ}=-\mathrm{RT}\ln K_{a}$$

where (*K*_a_)_1_, and (*K*_a_)_2_ are the binding constants at *T*_1_ and *T*_2_ and, *R* is the universal gas constant. If Δ*H*° < 0 and Δ*S*° < 0, van der Waals’ interactions and hydrogen bonds are dominant the binding reaction. If Δ*H*° > 0 and Δ*S*° > 0, hydrophobic interactions play a major role in the binding reaction. When Δ*H*° < 0 and Δ*S*° > 0, the electrostatic forces are more important than other forces (Ross and Subramanian, 1981). The thermodynamic parameter values for the binding interaction of BBR and BHB were tabulated in Table 5. The binding of BBR to BHB resulted in negative enthalpy (Δ*H*°) and positive entropy (Δ*S*°) values, which indicated that the electrostatic interaction was the major force in the binding reaction. The large positive entropy is the marker of the disruption and release of protein-bound condensed ions and water molecules (Basu & Suresh Kumar, 2015). In addition, the negative signal of Δ*G*° indicated that the binding process of BHB and BBR was spontaneous.

**Table 5.** The relative thermodynamic parameters of the BBR–BHB systems.

Sample	*T* (K)	*ΔH°* (kJ mol^−1^)	*ΔS°* (J mol^−1^K^−1^)	*ΔG°* (kJ mol^−1^)
BBR	293	−4.7495	72.113	−25.900
	298		72.114	−26.261
	310		71.724	−26.984

### 3.9. Investigation on BHB conformation changes

To investigate the effect of BBR on the conformation changes of BHB, UV-vis absorption spectra, synchronous fluorescence, circular dichroism and Raman spectra methods were utilized in the current study.

#### 3.9.1. UV–vis absorption spectra studies

**Figure 7.**
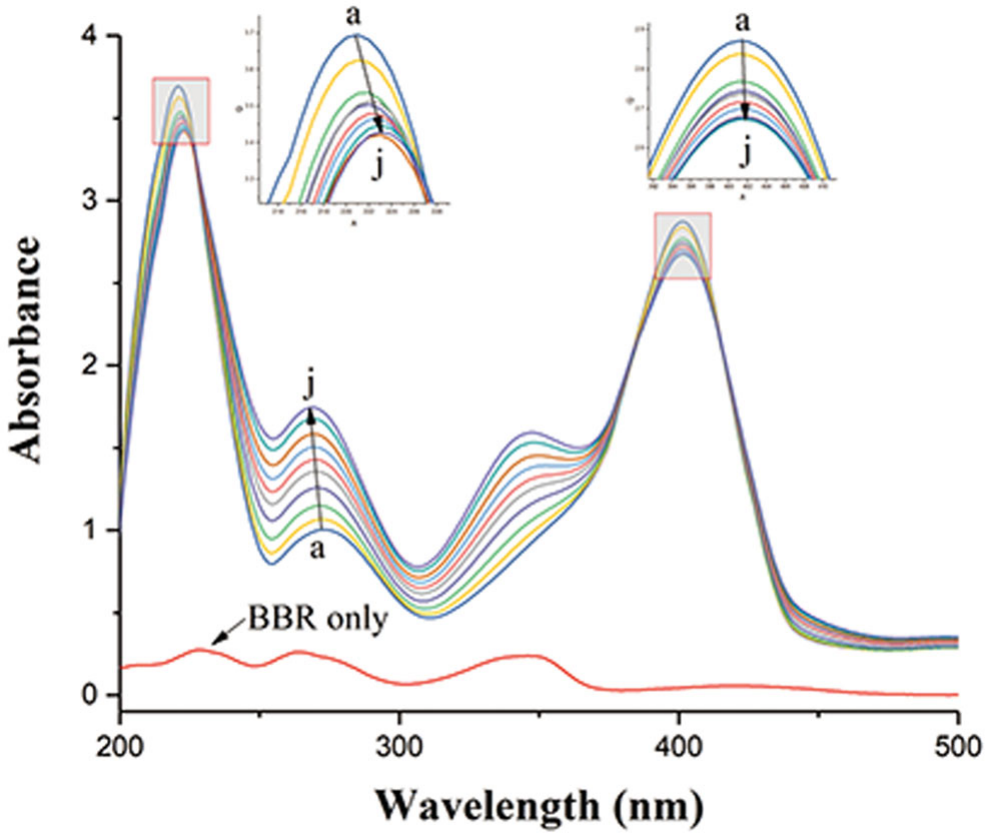
Absorption spectral titration of BHB (curve a) with increasing amounts of BBR (curves b–j). Conditions: BHB: 1 × 10^−5 ^mol/L; BBR/(10^−5 ^mol/L): (a) 0, (b) 0.2, (c) 0.4, (d) 0.6, (e) 0.8, (f) 1.0, (g) 1.2, (h) 1.4, (a) 1.6 and (j) 1.8. The concentration of BBR alone is 1.0 × 10^−5 ^mol/L; pH = 7.4 and T = 298 K.

The structural changes of protein and the protein-ligand complex formation can be measured by UV-vis absorption spectroscopy. As shown in Figure 7, there were three absorption peaks in BHB: a strong absorption peak at 210 nm reflecting the framework conformation of BHB, a weak absorption peak at 276 nm due to the aromatic amino acids (tryptophan, tyrosine and phenylalanine), and a strong peak at 405 nm attributed to the porphyrin-Soret band of BHB (Bao et al., 2001; Patil et al., 2007; Yang et al., 2009). The results showed that the intensity of the peak at 276 nm increased with blue shift and decreased with no shift at 210 and 405 nm after the gradual addition of BBR. These results indicated that the binding of BBR to BHB led to the change of protein skeleton and reduced the hydrophobicity of the microenvironment of the aromatic amino acid residues (Wu et al., 2007).

#### 3.9.2. Synchronous fluorescence

In order to better understand the conformational changes and microenvironment in the vicinity of the Trip or tyrosine (Tyr) residues of BHB. The synchronous fluorescence study was carried out in the current study. The shift (Δλ) of the wavelength of the fluorescence emission maximum reflects the polarity around the fluorophores in synchronous fluorescence spectra. When the shift of the wavelength of excitation and wavelength was set at 15 or 60 nm, the condition of tyrosine and tryptophan residues of BHB could be provided by synchronous fluorescence spectra (JBF, 1971).

**Figure 8.**
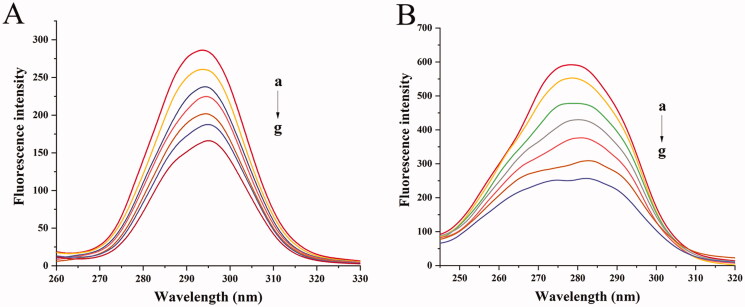
Synchronous fluorescence spectra of BHB with increasing amounts of BBR (curves b–g) (corrected): (A) Δ*λ* = 15 nm; (B) Δ*λ* = 60 nm. Conditions: BHB: 2 × 10^−6 ^mol/L; BBR (×10^−5^): (a) 0, (b) 0.25, (c) 0.5, (d) 0.75, (e) 1, (f) 1.25 and (g) 1.5; pH = 7.4 and *T* = 298 K.

There were three tryptophan (Trp) and five Tyr residues in BHB with a symmetry center. Figure 8(A) showed that the synchronous fluorescence of BHB with different concentrations of BBR, the gradual addition of BBR into BHB induced a red shift from 293.6 to 295 nm after gradual addition of BBR, which suggested that the hydrophobicity of the down-regulated Tyr residues migrated to a more hydrophilic environment (Klajnert and Bryszewska, 2002). In Figure 8(B), a red shift was observed from 278.4 to 282.2 nm, indicating that the addition of BBR had changed the molecular environment of Trp residue in BHB.

#### 3.9.3. Circular dichroism

The conformational changes of BHB had a strong relationship with the complexity of BBR. Therefore, the CD was used to probe the interaction. The secondary and tertiary structures of the proteins can be determined by CD spectroscopy (Khan and Kumar, 2016).

**Figure 9.**
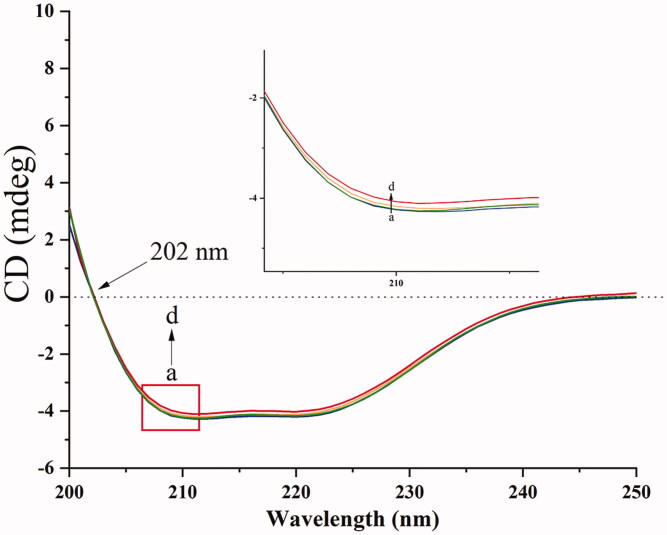
CD spectra of BHB and BHB-BBR systems. Conditions: BHB: 5.0 × 10^−8 ^mol/L; (a) 0, (b) 1 × 10^−7 ^mol/L, (c) 2 × 10^−7 ^mol/L and (d) 4 × 10^−7 ^mol/L; pH = 7.4 and *T* = 298 K.

In this work, the molar ratios of 1:0, 1:2, 1:4 and 1:8 for BHB: BBR were utilized for the CD records. The CD spectra of BHB at the absence (line a) or presence (lines b, c and d) of BBR were shown in Figure 9. The data of CD were expressed according to the following equation:

$$\mathrm{MRE}=\frac{{CD}_{\mathrm{obs}}(\mathrm{mdeg})}{C_{P}nl\times 10}$$

where MRE is the mean residue ellipticity (MRE) in deg cm^2^ dmol^−1^, CD_obs_ and C_P_ are the observed CD values and the molar concentration of protein, n and l are the number of amino acid residues (574) and the length of the cell (1 cm). Besides, the helix of α chain can be calculated following the below equation:

$$\alpha -\mathrm{Helix}(\%)=\frac{{-\mathrm{MRE}}_{208}-4000}{33000-4000}\times 100$$

where MRE_208_ is the observed MRE value at 208 nm, 4000 is the MRE value of β-form and random coil conformation cross at 208 nm, and 33,000 is the MRE value of pure α-helix at 208 nm (Shobini et al., 2001).

There are two minima at 208 and 222 nm in the native BHB between 190 and 250 nm in the far CD spectrum (Woody, 1995; Scarlett et al., 2015). Because the electronic transitions may upregulate the portion of the α-helical structure, the CD spectrum is expressed as negative minima data. Due to the π-π* transition, the minimum at 208 nm is the α-helical in the protein. Besides, n-π* transition exists in α-helical and random coil regions of the protein which resulted in a minima data at 222 nm. When BHB interacted with BBR of increasing concentration, the values of CD reduced significantly, which indicated that the secondary structure of BHB changed after the binding reaction. This phenomenon may be due to the unfolding α-helix which led to an extended chain of the polypeptide with increasing concentration of drugs. In brief, with an increment of BBR adding to BHB, the section of α-helicity reduced from 36.87% to 33.53%. It meant that BBR downregulated the content of α-helix in BHB after the interaction, suggesting that BBR bound to the amino acid residues of some major polypeptide chain and thus broke the balance of hydrogen bond networks (Lu et al., 2007; Shen et al., 2007). These mixtures may induce some conformational changes in BHB and affect the functions.

#### 3.9.4. Raman spectra

**Figure 10.**
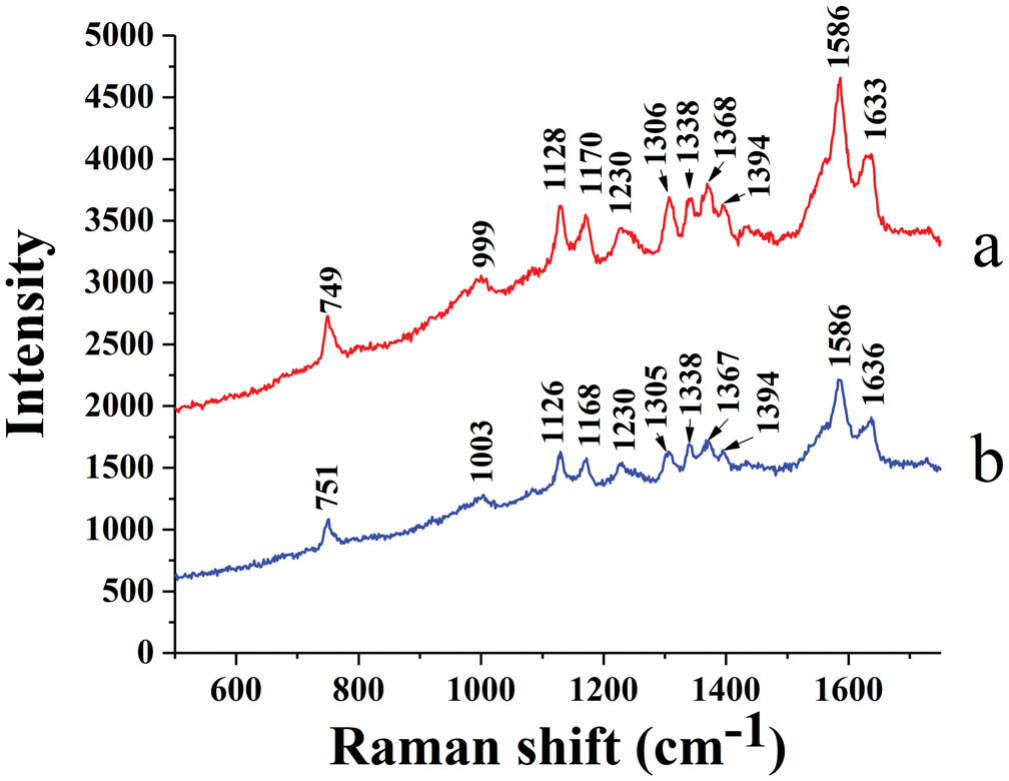
Raman spectrum of BHB (a) and BHB-BBR (b) system.

Raman spectra provide the molecular structure information and identify some chemical bonds. Figure 10 compares the Raman spectra of native BHB (Figure 10a) and BHB binding to BBR (Figure 10b). The signal at 1128 cm^−1^ is Cβ-methyl, the beta positions of porphyrins and pyrrole ring. The signal at 1170 cm^−1^ belongs to the pyrrole half-ring asymmetrical stretch (Atkins et al., 2017). The bands at 1338 and 1368 cm^−1^ are identified as the pyrrole half-ring symmetrical stretch (Li et al.,^1990^; Lu et al., 2007). These Raman vibrations correlate a response to the oxidation state of BHB Fe heme, and these signals are also sensitive to the quaternary structure of BHB (Nagai et al., 2018). Due to the excitation of laser or the sample heating (> 42 °C), the signal of BHB was identified at 1394 cm^−1^. These vibrational changes may be due to the aggregates of BHB which resulted from thermal or photo-induced protein denaturation (Wood et al., 2007; Lemler et al., 2014).

Moreover, the 1586 and 1633 cm^−1^ bands belong to the mode vibration of asymmetric stretching of Cα-Cm (alpha and meso positions of porphyrins and pyrrole ring) (Spiro, 1985; Li et al.,^1990^). The band of 1586 cm^−1^ is oxidation state sensitive, and the band of 1633 cm^−1^ is sensitive to the spin-state for Fe heme, both of these signals are characteristic bands for BHB in Raman spectra (Dybas et al., 2021). These stretching modes were observed to be slightly changed when binding with BBR (Figure 10), indicating that the spin state of the heme iron may be affected by the combinations.

As shown in Figure 10, the peak at 749 cm^−1^ belonged to Trp, while the intensity was reduced in the BHB-BBR system, indicating that BBR changed the microenvironment of Trp as the same in the previous fluorescence spectra. The band of 999 cm^−1^ belongs to the aromatic amino acid residues of phenyl modes in BHB such as phenylalanine (Phe), Trp, and Tyr (Venkatesh et al., 1999). After binding to BBR, the signals shifted to 1003 cm^−1^ which indicated that BBR affected those amino acid residues of phenyl modes. Based on the results of CD spectra (Figure 9) and synchronous fluorescence spectra (Figure 8), the interaction of BBR and BHB involved in the conformational changes of secondary and tertiary structure for the adsorbed BHB.

#### 3.9.5. Molecular docking and molecular dynamics simulation

**Figure 11.**
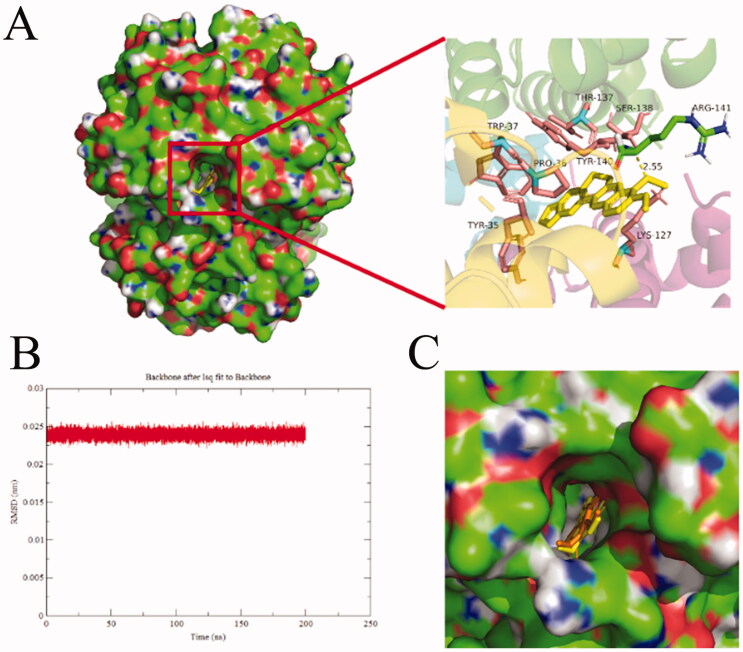
Docking conformations of BBR (A) in complex with BHB. (B) The rmsd value of BBR-BHB complex, (C) The protein-ligand conformation before (color: yellow) and after (color: orange) MD simulation. BBR was displayed in balls and sticks, yellow dotted lines indicated hydrogen bond (PDB ID: 1G09).

In order to further explore the binding site of BBR on BHB, the molecular docking experiment was carried out. After the docking search was completed, the conformers of the lowest binding energy conformers were searched out of 250 different types and the resultant one was utilized for further study. The lowest free energy for the BHB-BBR system was shown in Figure 11. Basically, the best-ranked results showed that BBR dye was surrounded by Trp-37, Thr-137, Pro-36, Tyr-35, Tyr-140, Ser-138, Arg-141 and Lys-127, respectively. BBR could interact with the Arg-141 residue of BHB, and the bond length was found to be 2.55 Å. The ΔG value of the BHB-BBR system was −31.79 kJ/mol by virtual computing.

After MD simulation, the value of the root mean square derivation (rmsd) of BBR-BHB was less than 0.025 nm, which indicated that the simulation generated a stable trajectory and the conformation was stable. The molecular docking and MD simulation results provided a better insight into the BHB-BBR system from the perspective of binding structural character.

## 4. Discussion

BBR has been considered as a promising drug candidate for metabolic disorders, cardiovascular disease and cancer for its favorable pharmacological activities. However, the contradiction between the limited plasma concentration and its broad therapeutic effect and promising prospects of BBR remained ambiguous. Our previous study has indicated that the low plasma concentration and pronounced bioactivities of BBR might be partially ascribed to the generation of its oxymetabolite oxyberberine (Chen et al., 2021), which exhibited superior anti-inflammatory (Li et al., 2019), anti-colitis (Li et al., 2020), anti-non-alcoholic fatty liver disease (Li et al., 2021), and hypoglycemic effects (Dou et al., 2021) with more favorable safety profile (Li et al., 2019). These endeavors might contribute to interpreting the pharmacokinetics and pharmacodynamics relationship of BBR from the perspective of metabolism. Our recent investigation has suggested that BBR exerted a much stronger binding interaction with hemoglobin than plasma, which was probably an important basis for the accumulation of BBR in blood (Chen et al., 2021). In the present work, the berberine erythrocyte-Hb self-assembly drug delivery system was hypothesized for the first time, which might contribute to further understanding the pharmacokinetics process of absorption, transport and release of BBR within the body.

In this study, we comparatively investigated the bioavailability of BBR via intravenous and oral administration and found that BBR had a low bioavailability (*C_0_* was 2.833 μg/mL after intravenous administration of 2.5 mg/kg BBR and the *C_max_* was 0.260 μg/mL post oral administration of 400 mg/kg BBR). Both of their *AUC_0–t_* values were found to be small, which was in agreement with other previous studies (Hua et al., 2007; Chen et al., 2011). Besides, as for the *t_1/2_*, the *t_1/2_* of BBR following intravenous or oral administration was 3.136 and 3.885 h, respectively. The close *t_1/2_* values after intravenous or oral administration of BBR might indicate that BBR could be affected directly by blood cells.

Numerous studies tried to explain the relationship between its pharmacokinetics and therapeutic effects from different aspects including solubility in the gastrointestinal tract, effective permeability coefficient and other physical and chemical properties, first-pass effect, liver enzyme, gastrointestinal metabolism and so on (Liu et al., 2016). However, these studies failed to fully explain the phenomenon of extremely low plasma concentration and high tissue distribution of BBR. The low bioavailability of BBR could be ascribed to the interaction of drugs with the blood cells and various proteins in the blood, which yielded the new direction of a self-assembled drug delivery system for our study. BBR has been reported to interact with proteins like serum albumin (Khan et al., 2013). However, the effects of other components of blood have been ignored, especially the erythrocytes, which account for about 45% of blood and constitute ca. 99% of all blood cell types (Klatt et al., 2020).

As a carrier, erythrocytes can make achievement of targeted drug delivery to enhance the therapeutic effects. Besides, among other advantages of the carrier, erythrocytes could improve the pharmacokinetic and pharmacodynamic properties of the drugs (Berikkhanova et al., 2016), and possess the properties of quickly identifying the targeted delivery and transporting to the target site (Fan et al., 2012a; 2012b). And studies on the application of erythrocytes as a slow drug release or site-targeted delivery system have been extensively performed (Hamidi et al., 2007; Fernandes et al., 2011). Hence, knowledge of erythrocyte partitioning of the compound is important to the interpretation and understanding of the pharmacokinetic profile and distribution of the compound.

The erythrocyte/plasma partition result indicated that the *C*_e/p_ values became smaller with the time of intravenous administration. The *C*_e/p_ value was close to ∞ after intravenous administration with BBR for 2 h, potentially indicating that most of BBR entered into erythrocyte and bound with Hb. Besides, BBR and erythrocytes tended to assemble rapidly *in vitro*, which might be due to the electrostatic attraction. From the structure of BBR, BBR has both hydrophobic and hydrophilic groups with a positive charge (Wang et al., 2016; Kostjukova and Kostjukov, 2021;; Patel, 2021), while the surface of erythrocytes has negative electrical potential (Fontes et al., 2008; Fernandes et al., 2011). Hence, the accumulation of BBR in erythrocytes might be intimately associated with electrostatic force, the potential primary step to form the BBR-erythrocyte system. The hemolysis rate indicated the BBR-erythrocyte system was relatively stable without hemolysis at the concentration of 1.00 mg/mL. And the maximum percentage of drug loading was 100% when the BBR-erythrocyte concentration was 0.185 μg/mL. Hence, 0.5 M (92.500 μg/mL) BBR was selected for the following experiments.

As for the transmission electron microscopy assay *in vitro*, erythrocytes treated with BBR were observed to be spheroidal with several intracellular vesicles when compared with normal erythrocytes. And it was speculated that BBR could be swallowed up by erythrocytes when it entered into the blood and came in contact with erythrocytes. As a self-assembled erythrocyte drug delivery system, drug loading into erythrocyte by endocytosis was preferable, since it has minimal effects on erythrocytes structure and morphology (Harisa et al., 2011). The incorporation of BBR into erythrocytes may occur by endocytosis, however, a further in-depth investigation was warranted. This finding indicated that BBR-erythrocyte could form a self-assembled drug carrier system naturally, which might provide an intriguing insight into the contradiction between the low bioavailability and the high tissue distribution of BBR. The above correlation may well be an indication of the significant role that erythrocyte plays in the storage and transport of BBR. BBR-erythrocyte self-assembled drug delivery system was envisaged to provide enlightenment to illuminate the pharmacological activity and therapeutic effect of BBR.

It is well-known that the pharmacological effect of a drug is dependent on both its pharmacokinetic and pharmacodynamic properties, which are largely influenced by the reversible binding of the drug to proteins in the blood (Schmidt et al., 2010; Borkar et al., 2015). The primary binding sites of drugs in erythrocytes are associated with Hb, proteins, or plasma membrane. Hb is the major (soluble) protein and makes up 97% of the erythrocyte’s dry weight. In addition to carrying O_2_ and CO_2_, Hb also has important physiological functions, such as storing endogenous metabolites and exogenous small molecules (Wang et al., 2007). And it is reported that Hb in blood plasma is naturally scavenged by monocytes and macrophages and is subsequently denatured in the lysosome, making it a natural drug carrier to target monocytes and macrophages (Zhang and Palmer, 2011; Singhal et al., 2017). In our work, the blood routine examination showed that the counts of WBC and PLT in blood did not fluctuate obviously, while the contents of erythrocyte and Hb were significantly reduced (*p* < .01 or *p* < .05) post intravenous administration of BBR, which might indicate the interaction of erythrocyte and Hb with BBR. Besides, Hb tended to accumulate in the liver, which was similar to the biodistribution of BBR (Ship et al., 2005; Tan et al., 2013), presumptively ascribed to the recognition and phagocytosis of BBR-loaded erythrocyte by the reticuloendothelial system of the liver. However, further endeavors are merited to provide definite insight.

The interacting mechanism of BBR binding to BHB was further explored. Results indicated BBR could bind with BHB and formed a BBR-BHB complex via a static quenching process. The binding mode and binding forces can be determined by the thermodynamic parameters. The binding of BBR to BHB resulted in negative enthalpy (Δ*H*° < 0) and positive entropy (Δ*S*° > 0) values, which indicated that the electrostatic interaction was the major force in the binding reaction. In addition, the negative signal of free energy change (Δ*G*° < 0) suggested that the binding process of BHB and BBR was spontaneous. Hence, the strong interaction of BBR binding to BHB was deduced to be a non-covalent bond.

Besides, to investigate the effect of BBR on the potential conformational changes of BHB, UV-vis absorption spectra, synchronous fluorescence, circular dichroism and Raman spectra methods were employed. BBR made strong interaction with BHB via binding in different positions and changed the microenvironment of aromatic amino acid residues in the UV and synchronous fluorescence study. The result of the circular dichroism spectrum showed that BBR exerted considerable effect in changing the α-helicity, and led to conformational changes when interacting with BHB. On the other hand, BBR changed the stretching mode of C_α_-C_m_ in BHB and made an influence on Trp in BHB in the Raman spectra. The molecular docking analysis suggested that BBR interacted with Arg-141 residue of BHB via hydrogen bond with the bond length of 2.55 Å. The ΔG value of the BHB-BBR system was −31.79 kJ/mol. Besides, molecular dynamics simulation showed that the rmsd of BBR-BHB was less than 0.025 nm, which indicated that the conformation was stable. This emphasized the role that Hb played in the interaction of BBR with erythrocytes. As shown in spectroscopy studies, molecular docking and molecular dynamics simulation, BBR was observed to bind to BHB, potentially contributing to facilitating the development of a BBR-Hb system for targeted drug delivery. The binding of BBR to Hb could conceivably play a role in mediating the transport of BBR *in vivo*.

A drug delivery system leads to a special function related to treating and preventing diseases by improving the bioavailability of the drug and its therapeutic efficacy. Associating a therapeutic molecule with a carrier may, however, result in the generation of immune actions against the carrier, hence the therapeutic drug delivery using natural biological carriers like erythrocytes is encouraging (Allen and Cullis, 2004). As for erythrocytes, macrophages play a pivotal role in erythrocytes production, maintenance and clearance. In the normal physiological condition, after BBR enters into erythrocytes, the drug-loaded erythrocytes could be processed by macrophages and the liver is an important erythrocytes depot, which has an important role in erythrocytes clearance (Lee et al., 2011; Theurl et al., 2016; Klei et al., 2017). Besides, the liver was reported to be the principal site of Hb uptake (Keene and Jandl, 1965; Nagel and Gibson, 1971). Therefore, both the process of macrophages and the erythrocytes clearance in the liver can “break down” the drug-deliver erythrocytes and release drugs. The process according with the extremely high concentration of BBR in the liver.

On the other hand, macrophages are not only critical components during erythroid maturation in a steady state but also during stress and disease. In a pathological state with inflammatory response, it seems that the “steady-state” macrophage populations are substituted for an inflammatory monocyte-derived pool of macrophages (Klei et al., 2017). Erythrocytes under pathological state tend to be phagocytosed by macrophages (Vos et al., 2011; Yin et al., 2021). In therapy, depending on the drug that is loaded, the erythrocytes can be used as carriers with a gradual drug release, as bioreactors or a system for targeted drug delivery (Pierigè et al., 2017). Therefore, in a pathological state, once BBR came in contact with erythrocytes, BBR and erythrocytes can act as a self-assembled drug delivery system, which can be processed by macrophages and delivered to targeted organs. During these processes mediated by macrophages, the self-assembled drug delivery system could be decomposed and BBR would be released into the targeted organs and tissues, facilitating its versatile pharmacological effects. However, more experiments should be performed to illuminate in more detail the involvement of macrophages in drug-loaded erythrocytes generation and release in normal and disease states.

Due to the unique biophysical properties, the erythrocytes-Hb drug delivery system is a great natural drug delivery system. Since the liver is the primary organ that supports rapid erythrocyte removal and Hb disposition (Theurl et al., 2016), the much higher concentrations of BBR in the liver probably could be explained by the underlying transport of the erythrocyte-Hb drug carrier system. Our results might provide supporting evidence of BBR binding to Hb as a basis for the accumulation and delivery of BBR in blood and high tissue distribution.

Taken together, BBR and erythrocytes-Hb could form a self-assembly system for drug delivery by interacting with erythrocytes and binding to Hb, which undoubtedly affected the pharmacokinetic and pharmacological action of BBR. BBR achieved higher concentrations in erythrocytes than in plasma; therefore, the conventional choice of plasma for bioanalysis might not be rational as compared to erythrocytes or whole blood. Erythrocytes might serve as a neglected compartment in the pharmacokinetics and pharmacodynamics of BBR, and the choice of the appropriate assay matrix for its pharmacokinetic analysis should be rationally based.

Our previous study has also provided a novel version to explain the contradiction between excellent pharmacological action and extremely low absolute bioavailability of BBR from the perspective of protein-bound drugs (Chen et al., 2021). Our current results indicative of a flow of BBR from erythrocytes to Hb was speculated to be an important transport mechanism of BBR, supporting the possibility that erythrocytes might play a pivotal role as a potentially effective way for the rapid distribution of circulating BBR to targeted organs. The results yielded a basis to illustrate the previously unsolved enigma of the pharmacokinetics of BBR and the extensive pharmacological action of BBR. However, there remains a need for further investigation to improve our understanding of the role of erythrocyte-Hb self-assembly delivery system playing in the transport and disposition of BBR and explore in further detail the involvement of macrophages in drug-loaded erythrocytes production and release.

## 5. Conclusion

In conclusion, our result indicated circulating erythrocyte might represent a reservoir of BBR and the strong binding affinity of BBR to Hb was responsible for the accumulation of BBR in the erythrocyte. The BBR erythrocyte-Hb self-assembled drug delivery system was proposed as a hidden carrier mode to provide a novel dimension to interpret the pharmacokinetics of BBR. Last but not least, the current study was an important step forward and also a new strategy to reevaluate the pharmacokinetics and pharmacological action of BBR, and provided enlightenment for the study on pharmacokinetics of other compounds using erythrocytes-Hb drug delivery system.
